# A global satisfaction degree method for fuzzy capacitated vehicle routing problems

**DOI:** 10.1016/j.heliyon.2022.e09767

**Published:** 2022-06-20

**Authors:** Juan Carlos Figueroa–García, Jhoan Sebastián Tenjo–García, Carlos Franco

**Affiliations:** a Universidad Distrital Francisco José de Caldas, Bogotá - Colombia; b Universidad Nacional de Colombia, Bogotá - Campus, Colombia; c Universidad del Rosario, Bogotá - Colombia

**Keywords:** Fuzzy optimization, Capacitated vehicle routing, Global satisfaction degree

## Abstract

There are several uncertain capacitated vehicle routing problems whose delivery costs and demands cannot be estimated using deterministic/statistical methods due to a lack of available and/or reliable data. To overcome this lack of data, third–party information coming from experts can be used to represent those uncertain costs/demands as fuzzy numbers which combined to an iterative–integer programming method and a global satisfaction degree is able to find a global optimal solution. The proposed method uses two auxiliary variables α,λ and the cumulative membership function of a fuzzy set to obtain real–valued costs and demands prior to find a deterministic solution and then iteratively find an equilibrium between fuzzy costs/demands via *α* and *λ*. The performed experiments allow us to verify the convergence of the proposed algorithm no matter the initial selection of parameters and the size of the problem/instance.

## 1 Introduction and state of the art

The *Vehicle Routing Problem* (VRP) is a popular class of combinatorial problems in logistics due to its applicability and relationship to last mile distribution where deterministic VRPs are among the most used in real world applications given the availability of methods to solve it (optimization, metaheuristics, etc.). Deterministic VRPs consider ideal parameters without uncertainty, but many applications are subject to different kinds of uncertainty which usually means to have a set of possible solutions which depends on the model/method to solve it. This way, uncertain VRPs (stochastic, interval–valued, fuzzy etc.) exhibit higher complexity, require extended models and specialized solution methods.

The *Capacitated Vehicle Routing Problem* (CVRP) is a subclass of VRPs (see Golden, Raghavan & Wasil [1], Braekers et al. [2] and Rocha et al. [3]) that includes customers, suppliers and also vehicles to shipping/transportation tasks (see Dantzig & Ramser [4], Christofides & Eilon [5] and Borčinová [6]) whose early versions consider deterministic costs/demands, so we refer to this problem as the *crisp* or just CVRP. Uncertain CVRPs have been addressed in different ways: Men, Jiang & Xu [7] solved a CVRP for transportation of hazardous materials with interval Type–2 fuzzy numbers, chance constrained programming and simulated annealing; Ewbank et al. [8] solved a fuzzy demands assignment problem using neural networks; Helal et al. [9] has solved a stochastic CVRP using a two–step method which combines a chance–constrained model and a stochastic model with recourse and Mańdziuk & Świechowski [10] solved a dynamic CVRP with random traffic jams using probabilistic upper bounds and decision trees to compare against ant-colony, tabu and evolutionary algorithms; Hannan et al. [11] used PSO algorithms to solid waste collection problems with uncertain transportation costs and environmental impact; Pekel & Kara [12] solved location routing problems with fuzzy demands and deterministic travel times using fuzzy chance constrained programming models; Wang et al. [13] solved a two-echelon CVRP with uncertain demands using genetic algorithms. M Shan–Huen [14] solved a multi-compartment capacitated location routing problem with stochastic demands and multiple–products using tabu search; Beraldi et al. [15] solved CVRPs with stochastic demands using a probabilistic formulation involving a predefined reliability degree and Thammano & Rungwachira [16] solved complex CVRPs by efficiently generating initial solutions via a sweep method evolved with ant colony algorithms to then be debugged/relinked using local search methods.

Sometimes the costs/demands of a CVRP lack of statistical data to be estimated, so third–party information coming from experts (represented as fuzzy sets) are a possible way to obtain reliable information. While previous works are focused to either uncertain costs or demands, this paper addresses CVRPs with delivering costs and demands affected by non–probabilistic uncertainty where information coming from experts represented as fuzzy sets is the main information source. This way, we extend the fuzzy iterative optimization algorithm proposed by Figueroa–García & Tenjo–García [17], Figueroa-García [18], and Figueroa-García & López-Bello [19], [20] who proposed an iterative Fuzzy Linear Programming (FLP) method to find a solution for fuzzy optimization problems with fuzzy technological parameters and continuous decision variables while the presented method solves a *Fuzzy Capacitated Vehicle Routing Problem* (FCVRP) which is a CVRP with fuzzy delivering costs, fuzzy demands and binary/integer decision variables i.e. a combinatorial problem.

The organization of the paper is as follows: Section 1 introduces the main topic; Section 2 presents the mathematical programming model of the CVRP; some basics on fuzzy numbers are presented in Section 3; Section 4 presents the fuzzy CVRP model and its solution method; Section 5 shows the performed experiments and Section 6 shows the final remarks of the paper.

## 2 Crisp/deterministic CVRP

A CVRP is a problem where a set of clients (*customers*) require goods (*demands*) which are sent from a set of sellers (*suppliers*) by using a transportation mean (*vehicle*) e.g. train, aircraft, ship, etc. Each vehicle is limited to a fixed/finite capacity which is usually not enough to cover all customers at once. In this problem, each customer is visited by a single vehicle starting from a *depot* (suppliers), covering a set of *nodes* (customers) to finally come back to the depot, limited to a finite amount of vehicles to cover all nodes in different *routes*. The aim of the CVRP is to minimize the total transportation cost of supplying all demands $d_{i}$ by covering each node by a single vehicle *x* using routes departing from a depot (node 0). Transportation costs are composed by all operational costs inherent to deliver the required demands using a vehicle. This way, an optimal CVRP minimizes the total delivering cost namely *z* as shown as follows.

**Index sets:**

i,j∈{0,1,2,⋯,m} is the set of origin–destination nodes (node 0 is the depot)

**Parameters:**

$c_{ij}\in R^{+}$ is the delivering cost to send a unit from the $i_{th}$ node to the $j_{th}$ node

$d_{i}\in R^{+}$ is the demand required by the $i_{th}$ node

K∈N is the availability of homogeneous vehicles

Q∈N is the capacity of a vehicle

**Decision variables:**

$x_{ij}\in \{0,1\}$ is the decision of a vehicle to traverse from the $i_{th}$ node to the $j_{th}$ node

$y_{ij}\in Z^{0+}$ is amount of supply to be sent from the $i_{th}$ node to the $j_{th}$ node

(1) $$\mathrm{Min}\;\;\sum_{i}\sum_{j,i\neq j}c_{ij}x_{ij},$$

(2) $$s.t.\sum_{i,i\neq j}x_{ij}=1\;\forall \;j\in \{1,2,\cdots ,m\},$$

(3) $$\sum_{j,i\neq j}x_{ij}=1\;\forall \;i\in \{1,2,\cdots ,m\},$$

(4) $$\sum_{i}x_{i0}⩽K,$$

(5) $$\sum_{j}x_{0j}⩽K,$$

(6) $$\sum_{i}x_{i0}-\sum_{j}x_{0j}=0,$$

(7) $$\sum_{j}y_{ji}-\sum_{j}y_{ij}=d_{i}\;\forall \;i\in \{1,2,\cdots ,m\},$$

(8) $$Q\cdot x_{ij}-y_{ij}⩾0\;\forall \;i,j\in \{1,2,\cdots ,m\},i\neq j,$$

(9) $$x_{ij}\in \{0,1\};\;\;y_{ij}⩾0.$$

**Figure 1:**
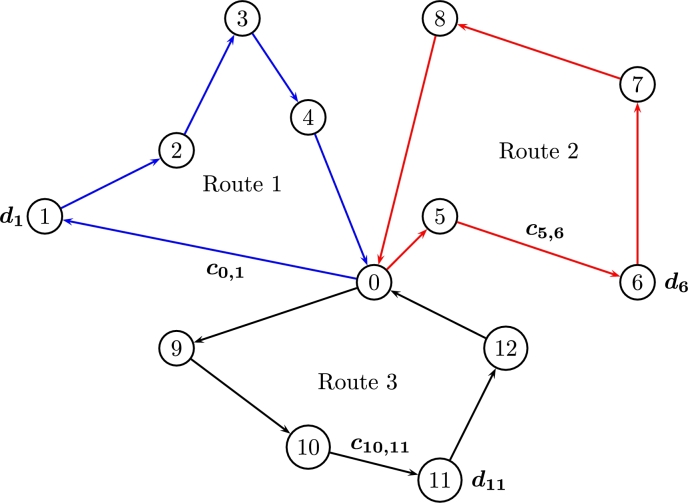
CVRP for three routes.

Fig. 1 shows and example of a CVRP of 12 nodes covered by three routes to/from a single depot.

CVRPs use deterministic time/distance units to define the cost of covering each customer (node) where each vehicle starts/ends from/to a *depot* (node 0). Eqs. (2) and (3) guarantee each customer *j* to be covered by a single vehicle. Eqs. (4), (5) guarantee not to send more than *K* available vehicles with equal capacity c∈R each. Eq. (6) guarantees each node to be covered once; Eq. (7) guarantees to satisfy required demands $d_{i}$, and Eq. (8) guarantees to send the required demands $y_{ij}$ by all nodes in a route. In general, the goal is to send all demands $d_{i}$ from i∈m origins to every j∈m destination using $y_{ij}$ in a single vehicle $x_{ij}$ with a capacity c∈R to cover the route.

## 3 Basics on fuzzy sets and numbers

A fuzzy set $\overset{˜}{A}=\{(x,\mu_{\overset{˜}{A}}(x))\;|\;x\in X\}$ is defined by a membership function $\mu_{\overset{˜}{A}}(x),x\in X$ which measures the membership of a value *x* regarding a concept/word/label *A*. F(X) is the class of all fuzzy sets, F(R) is the class of all real-valued fuzzy sets and $F_{1}(R)$ is the class of all fuzzy numbers. A fuzzy number $\overset{˜}{A}\in F_{1}(R)$ is then defined as follows.

Definition 1 Let $\overset{˜}{A}:R\to [0,1]$ be a fuzzy subset of the reals. Then $\overset{˜}{A}\in F_{1}(R)$ is a Fuzzy Number (FN) iff there exists a closed interval $[x_{l},x_{r}]\neq \emptyset$ with a membership function $\mu_{\overset{˜}{A}}(x)$ such that:

(10) $$\mu_{\overset{˜}{A}}(x)=\left\{ \begin{array}{cc} c(x)\; & \;\text{for}\;x\in [x_{l},x_{r}],\\ l(x)\; & \;\text{for}\;x\in [-\infty ,x_{l}],\\ r(x)\; & \;\text{for}\;x\in [x_{r},\infty ], \end{array}\right.$$

where c(x)=1 for $x\in [c_{l},c_{r}]$, $l:(-\infty ,x_{l})\to [0,1]$ is monotonic non-decreasing, continuous from the right, i.e. l(x)=0 for $x<x_{l}$; $l:(x_{r},\infty )\to [0,1]$ is monotonic non-increasing, continuous from the left, i.e. r(x)=0 for $x>x_{r}$.

The *α-cut* of a fuzzy number $\overset{˜}{A}\in F_{1}(R)$ namely A˜α≜{x|μA˜(x)⩾α}∀x∈X is then defined as follows:

(11) A˜α=[infx⁡μA˜α(x),supx⁡μA˜α(x)]=[aˇα,aˆα].

In probability theory, the cumulative probability function transforms any probability function into a monotonic non–decreasing measure which is very convenient in many cases. To do so, Figueroa-García & López-Bello [19], [20] and Figueroa-García [18] defined its fuzzy version as shown as follows.

Definition 2 **Cumulative Membership Function**

Let $\overset{˜}{A}\in F(X)$ be a fuzzy set. The Cumulative Membership Function (CMF) of $\overset{˜}{A}$, $\psi_{\overset{˜}{A}}(x)$ is:

(12) $$\psi_{\overset{˜}{A}}(x)≜Ps_{\overset{˜}{A}}(X⩽x)=\int_{-\infty }^{x}\mu_{\overset{˜}{A}}(t)\;dt.$$

Eq. (12) is the cumulative possibility of all X⩽x to occur regarding the linguistic label $\overset{˜}{A}$. Then $\psi_{\overset{˜}{A}}(x)$ can be normalized by the cardinality (or total area) of $\overset{˜}{A}$ namely $\left|\overset{˜}{A}\right|$, as follows:

(13) $${\overset{¯}{\psi }}_{\overset{˜}{A}}(x)≜\frac{1}{\left|\overset{˜}{A}\right|}\int_{-\infty }^{x}\mu_{\overset{˜}{A}}(t)\;dt=\frac{\int_{-\infty }^{x}\mu_{\overset{˜}{A}}(t)\;dt}{\int_{-\infty }^{\infty }\mu_{\overset{˜}{A}}(t)\;dt}$$

**Figure 2:**
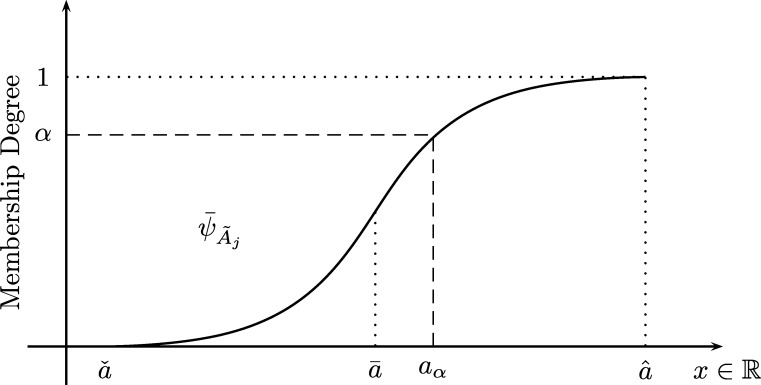
Normalized CMF of a fuzzy number $\overset{˜}{A}$.

Fig. 2 presents the normalized CMF of a fuzzy number i.e. ${\overset{¯}{\psi }}_{\overset{˜}{A}}$.

## 4 A proposal for solving FCVRPs

The FCVRP addressed in this paper fits into the family of FLPs that can be solved by the *Soft Constraints Method* (SCM) which was proposed by Zimmermann [21] & Verdegay [22], so we first introduce the SCM to then present an iterative SCM to solve FCVRPs.

### 4.1 Soft constraints method

Some approaches to solve/model fuzzy optimization problems were proposed by Sakawa et al. [23], Chanas et al. [24], Herrera & Verdegay [25], Peidro et al. [26], Najafi et al. [27] and Pishvaee & Khalaf [28] solved the problem $\mathrm{Min}\{\overset{˜}{z}=\overset{˜}{c}x:\overset{˜}{A}x≳\overset{˜}{b},x\in R^{+}\}$ using the Yager index [29] for $\overset{˜}{A}$; Donga & Wan [30] simplified $\overset{˜}{A}$ by using the fuzzy mean-value and Rena et al. [31] used a bi–level approach. All of them defuzzify all uncertain parameters prior to solve an LP while the presented method deals with fuzzy costs and demands at once via a satisfaction degree.

**Figure 3:**
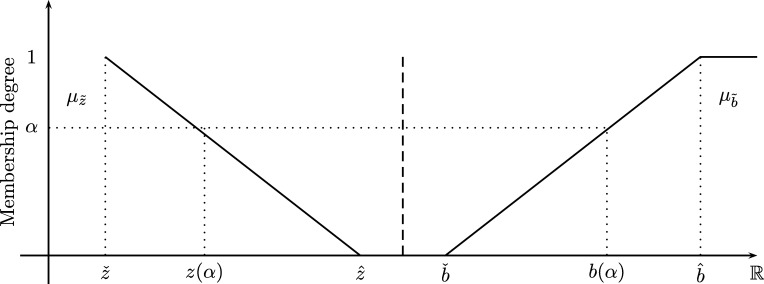
Shapes for $\overset{˜}{z}$ and $\overset{˜}{b}$.

The SCM solves problems in the form $\mathrm{Min}\{\overset{˜}{z}=c^{\prime }x:Ax≳\overset{˜}{b},x\in R^{+}\}$ whose constraints $\overset{˜}{b}$ are fuzzy linear sets as shown in Fig. 3 (right side) and A,c are crisp parameters. Its main goal is to maximize a global satisfaction degree namely *λ* between the goal $\overset{˜}{z}$ and the set of constraints $\overset{˜}{b}$ via the following LP:

(14) $$\underset{x,\lambda }{\mathrm{Max}}\;\;\lambda ,\text{s.t.}\;c^{\prime }x+\lambda (\overset{ˆ}{z}-\overset{ˇ}{z})=\overset{ˆ}{z},Ax-\lambda (\overset{ˆ}{b}-\overset{ˇ}{b})⩾\overset{ˇ}{b},x\in R^{+}$$

where λ∈[0,1], $\overset{ˇ}{z}={\mathrm{Min}}_{x}\{c^{\prime }x:Ax⩾\overset{ˇ}{b},x\in R^{+}\}$, $\overset{ˆ}{z}={\mathrm{Min}}_{x}\{c^{\prime }x:Ax⩾\overset{ˆ}{b},x\in R^{+}\}$ and the binary relation ≲ for fuzzy sets has been defined by Ramík and R̆imánek [32].

### 4.2 The fuzzy CVRP

The mathematical programming model for a FCVRP with fuzzy delivering/shipping costs, fuzzy demands and a limited/finite amount of vehicles is described as follows.

**Index sets:**

i,j∈{0,1,2,⋯,m} is the set of origin–destination nodes (node 0 is the depot)

**Parameters:**

${\overset{˜}{c}}_{ij}\in F_{1}(R^{+})$ is the fuzzy delivering cost to send a unit from the $i_{th}$ node to the $j_{th}$ node

${\overset{˜}{d}}_{i}\in F_{1}(R^{+})$ is the fuzzy demand required by the $i_{th}$ node

K∈N is the availability of homogeneous vehicles

Q∈N is the capacity of a vehicle

**Decision variables:**

$x_{ij}\in \{0,1\}$ is the decision of a vehicle to traverse from the $i_{th}$ node to the $j_{th}$ node $y_{ij}\in Z^{0+}$ is amount of supply to be sent from the $i_{th}$ node to the $j_{th}$ node

(15) $$\mathrm{Min}\;\;\sum_{i}\sum_{j,i\neq j}{\overset{˜}{c}}_{ij}x_{ij},$$

(16) $$s.t.\sum_{i,i\neq j}x_{ij}=1\;\forall \;j\in \{1,2,\cdots ,m\},$$

(17) $$\sum_{j,i\neq j}x_{ij}=1\;\forall \;i\in \{1,2,\cdots ,m\},$$

(18) $$\sum_{i}x_{i0}⩽K,$$

(19) $$\sum_{j}x_{0j}⩽K,$$

(20) $$\sum_{i}x_{i0}-\sum_{j}x_{0j}=0,$$

(21) $$\sum_{j}y_{ji}-\sum_{j}y_{ij}≳{\overset{˜}{d}}_{i}\;\forall \;i\in \{1,2,\cdots ,m\},$$

(22) $$Q\cdot x_{ij}-y_{ij}⩾0\;\forall \;i,j\in \{1,2,\cdots ,m\};i\neq j,$$

(23) $$x_{ij}\in \{0,1\};\;\;y_{ij}⩾0.$$

**Figure 4:**
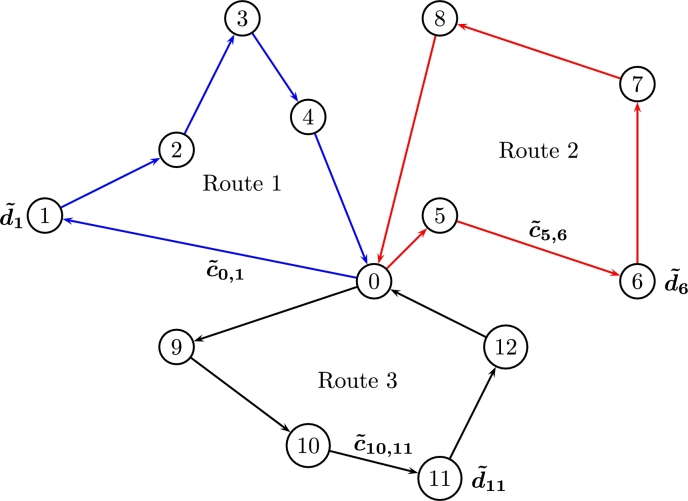
Fuzzy CVRP for three routes.

Eq. (15) is the total delivering cost given uncertain demands defined by experts as fuzzy sets (see Definition 1), all of them covered by a single vehicle $x_{ij}$ in the route (see Eq. (16) and Eq. (17)). In general, the FCVRP considers uncertain delivering costs ${\overset{˜}{c}}_{ij}$ defined as fuzzy numbers in order to cover uncertainties like climate, transportation times, road conditions, etc. that affect delivering tasks, and uncertain demands ${\overset{˜}{d}}_{i}$ which usually contain uncertainty induced by customers requirements, markets volatility, etc. Fig. 4 shows an FCVRP for three routes covering 12 nodes from the depot (node 0).

**Figure 5:**
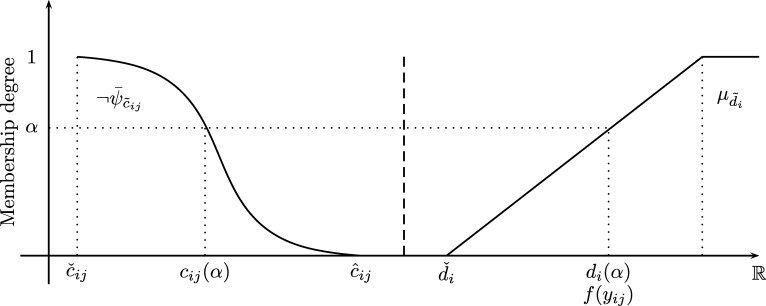
Shapes for ${\overset{˜}{c}}_{ij}$ and ${\overset{˜}{d}}_{i}$.

Fig. 4 involves uncertain demands $\overset{˜}{d}$ and delivering costs $\overset{˜}{c}$ which are defined as fuzzy numbers. In order to be able to use the SCM, we define $\overset{˜}{d}$ as a linear fuzzy constraint (see Fig. 5) as shown as follows.

Definition 3 Let us define the membership function of the fuzzy constraint (21) as follows:

(24) $$\mu_{{\overset{˜}{d}}_{i}}(f(y_{ij}),{\overset{ˇ}{d}}_{i},{\overset{ˆ}{d}}_{i})≜\left\{ \begin{array}{cc} 1, & f(y_{ij})⩾{\overset{ˆ}{d}}_{i}\\ \frac{f(y_{ij})-{\overset{ˇ}{d}}_{i}}{{\overset{ˆ}{d}}_{i}-{\overset{ˇ}{d}}_{i}}, & {\overset{ˇ}{d}}_{i}⩽f(y_{ij})⩽{\overset{ˆ}{d}}_{i}\\ 0, & f(y_{ij})⩽{\overset{ˇ}{d}}_{i} \end{array}\right.$$

where $f(y_{ij})=\sum_{j}y_{ji}-\sum_{j}y_{ij}$, ${\overset{ˇ}{d}}_{i}\in R_{+}$ and ${\overset{ˆ}{d}}_{i}\in R_{+}$ are the lower/upper bounds of ${\overset{˜}{d}}_{i}$.

Note that fuzzy demands as written in Eq. (21) are required to have a linear membership function (see Definition 3) since it allows us to make the equivalence between its left side $f(y_{ij})$ and the *α*–cut of ${\overset{˜}{d}}_{i}$ i.e.

$$\underset{f(y_{ij})}{\underset{︸}{\sum_{j}y_{ji}-\sum_{j}y_{ij}}}≳\;{\overset{˜}{d}}_{i}\Rightarrow f(y_{ij})\equiv \;d_{i}(\alpha )\to \frac{f(y_{ij})-{\overset{ˇ}{d}}_{i}}{{\overset{ˆ}{d}}_{i}-{\overset{ˇ}{d}}_{i}}=\alpha .$$

Definition 4 Let us define the set of all crisp constraints of the FCVRP i.e. Eqs. (16), (17), (18), (19), (20), (22) as $g(x_{ij})⩽,=b$ where *b* is the vector of crisp right hand side parameters.

In this paper we consider an FCVRP where $\overset{˜}{c}$ are fuzzy costs with any membership function and $\overset{˜}{d}$ with linear membership function (see Eqs. (10), (24) and Fig. 5). Now, to obtain a monotonic decreasing function of $\overset{˜}{c}$ which allows to obtain smaller values of $c_{ij}$ as *α* increases, we use the complement of ${\overset{¯}{\psi }}_{{\overset{˜}{c}}_{ij}}$.

Definition 5 Let ${\overset{˜}{c}}_{ij}$ be a fuzzy cost (see Eq. (10)) whose normalized CMF ${\overset{¯}{\psi }}_{{\overset{˜}{c}}_{ij}}$ (see Eq. (13)) is monotonic increasing and its complement $\neg {\overset{¯}{\psi }}_{{\overset{˜}{c}}_{ij}}:=1-{\overset{¯}{\psi }}_{{\overset{˜}{c}}_{ij}}$ is monotonic decreasing, then:

$$\alpha ↑\;\Rightarrow {\overset{¯}{\psi }}_{{\overset{˜}{c}}_{ij}}↑\;,c_{ij}↑,\alpha ↑\;\Rightarrow \neg {\overset{¯}{\psi }}_{{\overset{˜}{c}}_{ij}}↓\;,c_{ij}↓.$$

Also note that it is convenient to use the complement of the normalized CMF of ${\overset{˜}{c}}_{ij}$ i.e. $\neg {\overset{¯}{\psi }}_{{\overset{˜}{c}}_{ij}}=1-{\overset{¯}{\psi }}_{{\overset{˜}{c}}_{ij}}$ since it is monotonic decreasing and minimization problems look for minimum costs (the lower the better) while satisfying constraints (the bigger the better) at once as shown in Fig. 5.

### 4.3 The proposed method

Fuzzy demands $\overset{˜}{d}$ have linear shapes that represents soft inequalities in the form ≳ where $f(y_{ij})$ is the universe of discourse of ${\overset{˜}{d}}_{i}$ (see Eq. (24) and Fig. 5). On the other hand, fuzzy costs $\overset{˜}{c}$ can have any shape which are represented by $\neg {\overset{¯}{\psi }}_{\overset{˜}{c}}$ (see Definition 5 and Fig. 5) since demands and costs are in conflict of interest which can be written as the following decision making statement namely *D*:

D:The optimal routing decision is to covermaximum demandsat aminimum cost

**Figure 6:**
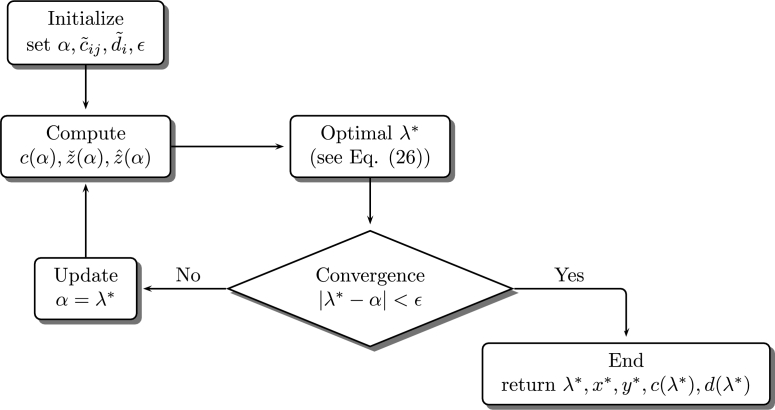
Flowchart of the proposed algorithm to solve FCVRPs.

The goal is to minimize fuzzy delivering costs $\sum_{i}\sum_{j,i\neq j}{\overset{˜}{c}}_{ij}x_{ij}$ subject to fuzzy demands ${\overset{˜}{d}}_{i}$. To do so, an iterative version of the SCM to solve the FCVRP is proposed and explained in Fig. 6.

Fig. 6 displays the flowchart of the proposed method which is divided into the following components: *initialize* by choosing a value *α*, defining fuzzy costs, demands and the admissible error *ϵ*, then a second step *computes* values $c(\alpha ),\overset{ˇ}{z}(\alpha ),\overset{ˆ}{z}(\alpha )$ needed to obtain the *optimal* value of the SCM namely $\lambda^{⁎}$ which is required to evaluate *convergence* of the method by computing $\left|\lambda^{⁎}-\alpha \right|$ so if it is lesser than *ϵ* then the algorithm stops, but if $\left|\lambda^{⁎}-\alpha \right|$ is greater than *ϵ* then *α* is *updated* with the obtained $\lambda^{⁎}$, and the process is repeated until $\lambda^{⁎}\approx \alpha$ i.e. $|\lambda^{⁎}-\alpha |<\epsilon$ which is the point where $d(\lambda^{⁎}),z(\lambda^{⁎})$ are in equilibrium with c(α). Now, the full description of the proposed algorithm based on the SCM (see Eq. (14), Figueroa–García & Tenjo–García [33], Figueroa–García & Tenjo–García [17], Figueroa-García [18] and Figueroa-García & López-Bello [19], [20]) is as follows.

Algorithm 1 **1- Setup:**

– Set α∈[0,1],

– Compute ${\overset{¯}{\psi }}_{{\overset{˜}{c}}_{ij}}$ and $c_{ij}(\alpha )=\neg {\overset{¯}{\psi }}_{{\overset{˜}{c}}_{ij}}^{-1}(\alpha )\;\forall \;i,j\in \{0,1,\cdots ,m\}$,

**2- The soft constraints method:**

– Compute $\overset{ˇ}{z}(\alpha )=\mathrm{Min}\{\sum_{i}\sum_{j,i\neq j}c_{ij}(\alpha )x_{ij}:g(x_{ij})⩽,=b;\sum_{j}y_{ji}-\sum_{j}y_{ij}⩾{\overset{ˇ}{d}}_{i}\}$

– Compute $\overset{ˆ}{z}(\alpha )=\mathrm{Min}\{\sum_{i}\sum_{j,i\neq j}c_{ij}(\alpha )x_{ij}:g(x_{ij})⩽,=b;\sum_{j}y_{ji}-\sum_{j}y_{ij}⩾{\overset{ˆ}{d}}_{i}\}$

– Define the fuzzy set $\overset{˜}{z}(\alpha )$ with a membership function (see Fig. 3):

(25) $$\mu_{\overset{˜}{z}}(z,\overset{ˇ}{z},\overset{ˆ}{z}\;|\;\alpha )=\left\{ \begin{array}{cc} 1, & z(\alpha )⩽\overset{ˇ}{z}(\alpha )\\ \frac{\overset{ˆ}{z}(\alpha )-z(\alpha )}{\overset{ˆ}{z}(\alpha )-\overset{ˇ}{z}(\alpha )}, & \overset{ˇ}{z}(\alpha )⩽z(\alpha )⩽\overset{ˆ}{z}(\alpha )\\ 0, & z(\alpha )⩾\overset{ˆ}{z}(\alpha ) \end{array}\right.$$

where $z(\alpha )=\sum_{i}\sum_{j,i\neq j}c_{ij}(\alpha )\;x_{ij}$.

– Thus, solve the following LP model:

(26) $$\underset{x,y,\lambda }{\mathrm{Max}}\;\;\lambda ,$$

(27) $$s.t.\sum_{i}\sum_{j,i\neq j}c_{ij}(\alpha )\;x_{ij}+\lambda (\overset{ˆ}{z}(\alpha )-\overset{ˇ}{z}(\alpha ))=\overset{ˆ}{z}(\alpha ),$$

(28) $$g(x_{ij})⩽,=b,$$

(29) $$\sum_{j}y_{ji}-\sum_{j}y_{ij}-\lambda ({\overset{ˆ}{d}}_{i}-{\overset{ˇ}{d}}_{i})⩾{\overset{ˇ}{d}}_{i}\;\forall \;i\in \{1,2,\cdots ,m\},x_{ij}\in \{0,1\},\;y_{ij}⩾0,\;\lambda \in [0,1].$$

**3- Convergence:**

– If $\lambda^{⁎}=\alpha$ then stop and return $\lambda^{⁎}$ as the overall satisfaction degree of $\overset{˜}{z},{\overset{˜}{c}}_{ij}$, and ${\overset{˜}{d}}_{i}$; if $\lambda^{⁎}\neq \alpha$ then go to Step 1 and update $\alpha =\lambda^{⁎}$.

Note that the set $\overset{˜}{z}(\alpha )$ is a function of *α* i.e. $z(\alpha )=\sum_{i}\sum_{j,i\neq j}c_{ij}(\alpha )x_{ij},\alpha \in [0,1]$ that represents the *minimum delivering cost* with a highest value given by $\overset{ˇ}{z}(\alpha )$ and a lowest value given by $\overset{ˆ}{z}(\alpha )$ for a given α∈[0,1]. On the other hand, the demands $\overset{˜}{d}$ get its highest value at $\overset{ˆ}{d}$ and its lowest value at $\overset{ˇ}{d}$. Also note that the Zimmermann's method maximizes the overall satisfaction degree between $\overset{˜}{z}(\alpha )$ (through $\overset{˜}{c}(\alpha )=\neg {\overset{¯}{\psi }}_{\overset{˜}{c}}^{-1}(\alpha )$) and $\overset{˜}{d}$ through an auxiliary variable *λ*. It is important to remark that *α* is a variable that returns crisp values of $\overset{˜}{c}$ which are required to solve the SCM till $\lambda^{⁎}=\alpha$ iteratively where *λ* is the overall satisfaction degree between $\overset{˜}{z}(\alpha )$ and $\overset{˜}{d}$ (see Fig. 5).

### 4.4 Other approaches

The FCVRP has been addressed in the literature mostly using ranking measures for its fuzzy parameters which is a simplified deterministic solution. For instance, Zulvia, Kuo & Hu [34] proposed a method for solving a CVRP with ranked fuzzy travel times, demands and credibility measures; a similar problem was addressed by Brito et al. [35] by using a metaheuristic based in local search procedures; Kuo, Wibowo & Zulvia [36] solved a dynamic CVRP with fuzzy service times using ant colony optimization and Singh, Sharma & Chakraborty [37], [38] handle fuzzy demands through a mixed fuzzy ranking/stochastic approach while our proposal keep fuzzy information via its cumulative membership function and a global satisfaction degree unlike the above approaches which solve a ranking–based instance of the FCVRP.

## 5 Application example

To illustrate how to solve FCVRPs using the proposed algorithm, 8 different instances were taken from https://www.coin-or.org/SYMPHONY/branchandcut/VRP/data/index.htm and tested using crisp methods (see Borčinová [6]) and the proposed method (see Section 4.3). First, we solve the crisp subset P-n016-k08 of the Set P composed by i,j∈{0,1,⋯,14} nodes to cover with 8 vehicles with capacity c=35 to then solve the FCVRP for a mix of triangular T(a,b,c) and Gaussian G(μ,δ) fuzzy costs ${\overset{˜}{c}}_{ij}$ (triangular and Gaussian are popular shapes in practical applications) and demands ${\overset{ˇ}{d}}_{i},{\overset{ˆ}{d}}_{i}$ (see Table 3 in the Appendix). Table 2 presents a summary report of the obtained results for the 8 selected instances.

### 5.1 Crisp solution

The instance P-n016-k08 (see Augerat et al. [39]) is composed by the following costs/demands:

$$c=\left[\begin{array}{cccccccccccccccc} - & \; & \; & \; & \; & \; & \; & \; & \; & \; & \; & \; & \; & \; & \; & \\ 14 & -\; & \; & \; & \; & \; & \; & \; & \; & \; & \; & \; & \; & \; & \; & \\ 21 & 12\; & -\; & \; & \; & \; & \; & \; & \; & \; & \; & \; & \; & \; & \; & \\ 33 & 19\; & 15\; & -\; & \; & \; & \; & \; & \; & \; & \; & \; & \; & \; & \; & \\ 22 & 12\; & 22\; & 21\; & -\; & \; & \; & \; & \; & \; & \; & \; & \; & \; & \; & \\ 23 & 24\; & 16\; & 31\; & 36\; & -\; & \; & \; & \; & \; & \; & \; & \; & \; & \; & \\ 12 & 12\; & 11\; & 25\; & 24\; & 13\; & -\; & \; & \; & \; & \; & \; & \; & \; & \; & \\ 22 & 19\; & 9\; & 23\; & 30\; & 8\; & 10\; & -\; & \; & \; & \; & \; & \; & \; & \; & \\ 32 & 21\; & 12\; & 8\; & 26\; & 25\; & 23\; & 18\; & -\; & \; & \; & \; & \; & \; & \; & \\ 32 & 27\; & 15\; & 24\; & 37\; & 13\; & 20\; & 10\; & 17\; & -\; & \; & \; & \; & \; & \; & \\ 21 & 7\; & 11\; & 12\; & 12\; & 26\; & 16\; & 19\; & 15\; & 25\; & -\; & \; & \; & \; & \; & \\ 28 & 19\; & 29\; & 25\; & 7\; & 43\; & 31\; & 37\; & 32\; & 44\; & 19\; & -\; & \; & \; & \; & \\ 30 & 16\; & 19\; & 9\; & 13\; & 35\; & 26\; & 28\; & 17\; & 31\; & 10\; & 16\; & -\; & \; & \; & \\ 29 & 21\; & 9\; & 17\; & 30\; & 16\; & 17\; & 9\; & 10\; & 7\; & 18\; & 37\; & 24\; & -\; & \; & \\ 31 & 33\; & 24\; & 37\; & 44\; & 8\; & 21\; & 15\; & 31\; & 16\; & 34\; & 51\; & 43\; & 21\; & -\; & \\ 30 & 17\; & 23\; & 16\; & 9\; & 39\; & 28\; & 32\; & 23\; & 37\; & 13\; & 10\; & 6\; & 30\; & 47\; & - \end{array}\right];d=\left[\begin{array}{c} 19\\ 30\\ 16\\ 23\\ 11\\ 31\\ 15\\ 28\\ 8\\ 8\\ 7\\ 14\\ 6\\ 19\\ 11 \end{array}\right]$$

**Figure 7:**
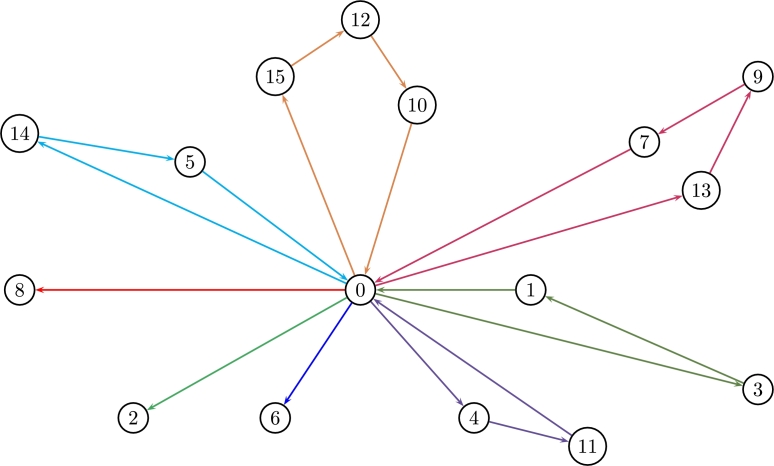
Crisp CVRP for 8 routes (instance P-n016-k08).

In this instance, delivering costs are deterministic distances with no uncertainty and each vehicle has a fixed capacity of c=35 units. The crisp solution (see Borčinová [6]) is $z^{⁎}=450$ covered by 8 routes i.e. 8 vehicles as displayed in Fig. 7.

$$\begin{array}{cccc} r_{1}:\;0-2-0\; & r_{2}:\;0-6-0\; & r_{3}:\;0-8-0\; & r_{4}:\;0-15-14-10-0\\ r_{5}:\;0-14-5-0\; & r_{6}:\;0-13-9-7-0\; & r_{7}:\;0-11-4-0\; & r_{8}:\;0-3-1-0 \end{array}$$

### 5.2 Fuzzy solution

Algorithm 1 can start with any *α* to then compute ${\overset{¯}{\psi }}_{{\overset{˜}{c}}_{ij}}$ and $c(\alpha )=\neg {\overset{¯}{\psi }}_{{\overset{˜}{c}}_{ij}}(\alpha )\;\forall \;i,j$ (see Definition 5). The optimal $\lambda^{⁎}=0.564429$ leads to $z(\lambda^{⁎})=461.09,\overset{ˇ}{z}(\lambda^{⁎})=416.79,\overset{ˆ}{z}(\lambda^{⁎})=518.48$ which is a bit more expensive than the crisp solution. The 8 optimal routes that cover all nodes are:

$$\begin{array}{cccc} r_{1}:\;0-1-7-0\; & r_{2}:\;0-2-0\; & r_{3}:\;0-4-11-0\; & r_{4}:\;0-5-14-0\\ r_{5}:\;0-6-0\; & r_{6}:\;0-8-0\; & r_{7}:\;0-9-13-3-0\; & r_{8}:\;0-10-12-15-0 \end{array}$$

**Figure 8:**
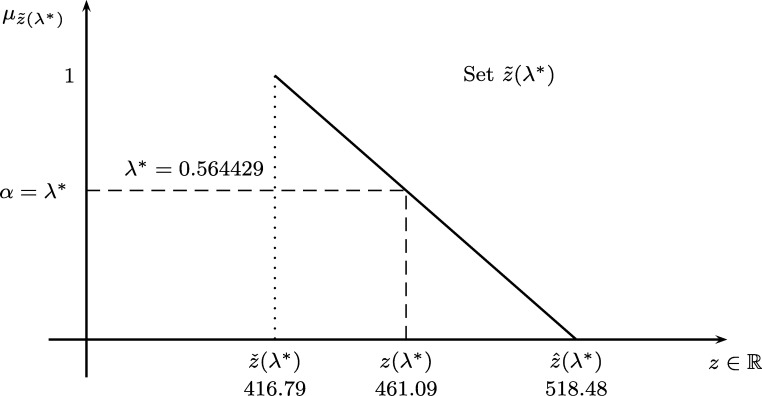
Optimal solution of the problem.

The proposed solution covers more demands ${\overset{˜}{d}}_{i}$ via $\lambda^{⁎}$ while finding the highest allowable costs ${\overset{˜}{c}}_{ij}$ via *α* and $\neg {\overset{¯}{\psi }}_{{\overset{˜}{c}}_{ij}}$ which means that the following system of equations (see Eqs. (27), (28) and (29)):

$$\sum_{i}\sum_{j,i\neq j}c_{ij}(\alpha )x_{ij}+\lambda^{⁎}(\overset{ˆ}{z}(\lambda^{⁎})-\overset{ˇ}{z}(\lambda^{⁎}))=\overset{ˆ}{z}(\lambda^{⁎}),g(x_{ij})⩽,=b,\sum_{j}y_{ji}-\sum_{j}y_{ij}-\lambda^{⁎}({\overset{ˆ}{d}}_{i}-{\overset{ˇ}{d}}_{i})⩾{\overset{ˇ}{d}}_{i}\;\forall \;i\in \{1,2,\cdots ,m\},x_{ij}\in \{0,1\},\;y_{ij}⩾0,\;\lambda^{⁎}⩽\alpha ⩽1$$

has a solution for any $\alpha ⩾\lambda^{⁎}\;\Rightarrow c_{ij}(\alpha )⩽c_{ij}(\lambda^{⁎})$ but it has no solution for $\alpha <\lambda^{⁎}\;\Rightarrow c_{ij}(\alpha )>c_{ij}(\lambda^{⁎})$. This is equivalent to say that the maximum allowable costs/demands of the FCVRP are $c_{ij}(\lambda^{⁎})$ and $d(\lambda^{⁎})=f(y_{ij})$ (see Definition 3, Definition 5), so cheaper costs and/or less demands than $\lambda^{⁎}$ are feasible too. This helps decision making since it provides a set of possible solutions instead of a deterministic solution. Fig. 8 shows the set $\overset{˜}{z}$ of optimal costs with a global optimal degree $\lambda^{⁎}=0.564429$.

**Figure 9:**
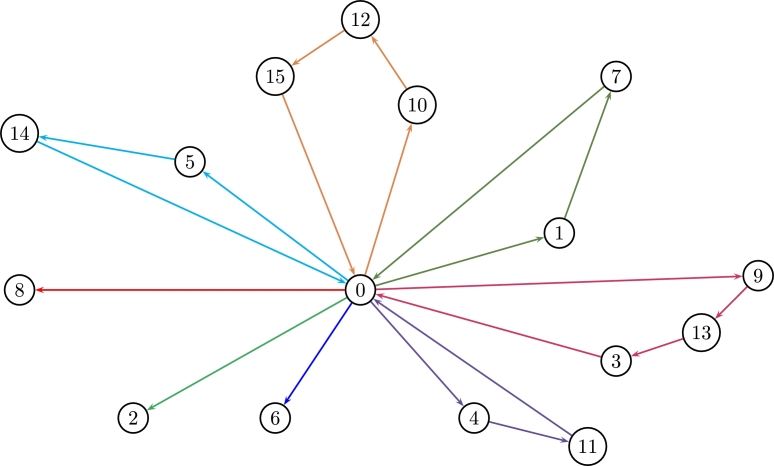
Optimal routes of the FCVRP for 8 vehicles (instance P-n016-k08).

The auxiliary variable *λ* looks for the equilibrium between quantities to be sent and its costs which define the total cost of the system, so the bigger $c_{ij}(\alpha )$ the lesser demands are covered at a total cost $z(\lambda^{⁎})$. Also note that $\lambda^{⁎}$ converges for any starting point of α∈[0,1]. Fig. 9 shows the 8 optimal routes for $\lambda^{⁎}$.

The defuzzified values $c(\lambda^{⁎})$ and $d(\lambda^{⁎})$ are as follows:

$$\left[\begin{array}{cccccccccccccccc} - & \; & \; & \; & \; & \; & \; & \; & \; & \; & \; & \; & \; & \; & \; & \\ 13.60 & -\; & \; & \; & \; & \; & \; & \; & \; & \; & \; & \; & \; & \; & \; & \\ 20.35 & 11.68\; & -\; & \; & \; & \; & \; & \; & \; & \; & \; & \; & \; & \; & \; & \\ 33.18 & 19.48\; & 14.68\; & -\; & \; & \; & \; & \; & \; & \; & \; & \; & \; & \; & \; & \\ 21.57 & 11.96\; & 21.73\; & 20.23\; & -\; & \; & \; & \; & \; & \; & \; & \; & \; & \; & \; & \\ 22.31 & 23.26\; & 15.73\; & 30.30\; & 35.01\; & -\; & \; & \; & \; & \; & \; & \; & \; & \; & \; & \\ 12.09 & 12.32\; & 10.56\; & 24.03\; & 23.46\; & 13.09\; & -\; & \; & \; & \; & \; & \; & \; & \; & \; & \\ 21.64 & 18.45\; & 9.32\; & 21.73\; & 30.48\; & 7.87\; & 10.25\; & -\; & \; & \; & \; & \; & \; & \; & \; & \\ 30.52 & 20.66\; & 12.09\; & 8.09\; & 25.87\; & 24.29\; & 22.60\; & 18.48\; & -\; & \; & \; & \; & \; & \; & \; & \\ 31.90 & 26.52\; & 14.56\; & 22.57\; & 36.16\; & 13.09\; & 19.56\; & 10.64\; & 17.80\; & -\; & \; & \; & \; & \; & \; & \\ 20.56 & 7.16\; & 10.29\; & 11.23\; & 11.54\; & 25.73\; & 15.74\; & 18.38\; & 15.64\; & 24.29\; & -\; & \; & \; & \; & \; & \\ 27.17 & 18.90\; & 27.85\; & 24.49\; & 6.62\; & 41.50\; & 30.05\; & 35.50\; & 31.27\; & 42.72\; & 18.17\; & -\; & \; & \; & \; & \\ 28.90 & 15.36\; & 18.62\; & 8.62\; & 12.56\; & 34.23\; & 24.90\; & 27.36\; & 16.57\; & 30.17\; & 9.87\; & 19.99\; & -\; & \; & \; & \\ 28.44 & 20.47\; & 9.32\; & 16.59\; & 29.34\; & 16.32\; & 17.71\; & 9.09\; & 10.56\; & 7.09\; & 17.31\; & 36.22\; & 23.28\; & -\; & \; & \\ 31.32 & 32.43\; & 23.09\; & 35.74\; & 42.82\; & 8.80\; & 20.57\; & 14.11\; & 29.99\; & 15.72\; & 33.06\; & 53.14\; & 41.88\; & 19.24\; & -\; & \\ 29.34 & 16.61\; & 22.80\; & 15.64\; & 8.93\; & 37.89\; & 26.47\; & 30.99\; & 22.43\; & 35.50\; & 13.41\; & 9.87\; & 6.16\; & 28.91\; & 13.00\; & - \end{array}\right];\left[\begin{array}{c} -\\ 17.90\\ 27.03\\ 16.77\\ 23.77\\ 11.52\\ 29.34\\ 17.34\\ 27.60\\ 10.64\\ 8.26\\ 9.08\\ 15.90\\ 9.08\\ 18.34\\ 13.21 \end{array}\right]$$

For instance, Table 1 shows all iterations $k,\overset{ˇ}{z}(\alpha ),\overset{ˆ}{z}(\alpha )$ and $\lambda^{⁎}$ for three α={0.1,0.5,0.9}.

**Table 1:** Behavior of Algorithm for different starting *α*.

*k*	Starting with α=0.1				Starting with α=0.5				Starting with α=0.9			
	$\overset{ˇ}{z}(\alpha )$	$\overset{ˆ}{z}(\alpha )$	*z*(*λ*^⁎^)	*λ*^⁎^	$\overset{ˇ}{z}(\alpha )$	$\overset{ˆ}{z}(\alpha )$	*z*(*λ*^⁎^)	*λ*^⁎^	$\overset{ˇ}{z}(\alpha )$	$\overset{ˆ}{z}(\alpha )$	*z*(*λ*^⁎^)	*λ*^⁎^
1	394.73	666.66	527.65	0.5112	405.64	527.79	460.57	0.5503	392.12	409.38	400.14	0.5354
2	404.45	526.17	459.19	0.5503	411.47	520.53	457.99	0.5734	405.64	522.68	457.44	0.5574
3	411.46	520.53	457.99	0.5734	415.69	517.18	459.90	0.5644	410.63	519.51	457.10	0.5732
4	415.69	517.18	459.90	0.5644	416.79	518.49	461.09	0.5644	415.71	517.2	459.93	0.5643
5	416.8	518.49	461.10	0.5644	416.79	518.49	461.09	0.5644	416.8	518.49	461.10	0.5644
6	416.79	518.49	461.09	0.5644	416.79	518.48	461.09	0.5644	416.79	518.49	461.09	0.5644
7	416.79	518.48	461.09	0.5644					416.79	518.48	461.09	0.5644

**Figure 10:**
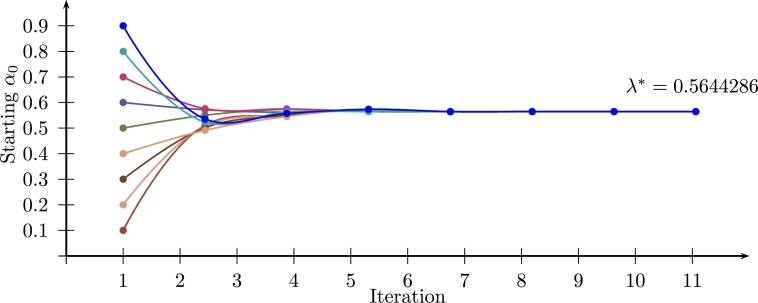
Iterative FCVRP for different *α*_0_ (instance P-n016-k08).

Fig. 10 shows $\lambda_{k}^{⁎}$ for 9 different starting values α={0.1→0.9}, all of them converge to $\lambda^{⁎}=0.564429$

Finally, we have tested the algorithm by solving the 8 instances presented by Borčinová [6]. The obtained results are shown in Table 2.

**Table 2:** Results of the CVRP and FCVRP for 8 different instances.

Instance	Crisp solution				Fuzzy solution				
	Cap.	Nodes	# Veh.	Sol.	$\overset{ˇ}{z}(\lambda^{⁎})$	$\overset{ˆ}{z}(\lambda^{⁎})$	*z*(*λ*^⁎^)	*λ*^⁎^	Time (hours)
P-n16-k8	35	15	8	450	416.79	518.48	461.08	0.564429	3.146
P-n19-k2	160	18	2	212	207.34	218.78	212.69	0.532487	3.468
P-n20-k2	160	19	2	216	199.37	228.32	210.56	0.61354	3.702
P-n21-k2	160	20	2	211	201.35	220.18	209.47	0.56872	3.949
P-n22-k2	160	21	2	216	203.18	231.48	215.32	0.57113	4.534
En13-k4	6000	12	4	247	222.35	267.58	240.93	0.58924	2.512
E-n22-k4	6000	21	4	375	371.98	401.45	384.56	0.573217	5.136
E-n23-k3	4500	22	3	569	562.83	583.71	570.69	0.62375	4.682

We recall that all instances converge to a single $\lambda^{⁎}$ which is the global equilibrium degree between fuzzy costs and satisfaction of the demands. The crisp CVRP is convenient only for deterministic conditions, but it has no flexibility to cover uncertain demands as the presented approach does since $\lambda^{⁎}$ is the maximum degree in which demands can be covered at minimum cost and $\overset{ˇ}{z}(\lambda^{⁎}),\overset{ˆ}{z}(\lambda^{⁎})$ are the costs associated to cover minimum/maximum demands $\overset{ˇ}{d}$ and $\overset{ˆ}{d}$. For instance, if we compute the total cost of the optimal crisp solution using $c(\lambda^{⁎})$ we obtain $z^{⁎}=443.71$ which is cheaper than the pure deterministic solution.

## 6 Concluding remarks

The algorithm proposed by Figueroa-García [18] and Figueroa-García & López-Bello [19], [20] have been extended to solve FCVRPs with satisfactory results. The optimal satisfaction degree $\lambda^{⁎}$ is a global defuzzifier for all fuzzy parameters $\overset{˜}{z}$, ${\overset{˜}{c}}_{ij},{\overset{˜}{d}}_{i}$ and it gets the maximum allowable costs and demands to be satisfied by the system while holding feasibility. The provided examples/instances illustrate the way how the iterative method solves FCRVPs whose results in the 8 selected instances show convergence to a global optimal $\lambda^{⁎}$ and subsequently to optimal values $z,c_{ij}$ and $d_{i}$ with better results in some instances.

The proposed algorithm deals with nonlinear fuzzy delivering costs and fuzzy demands by iterating the SCM to then obtain a maximum $\lambda^{⁎}$ which is the global optimal satisfaction degree between costs and demands, so any $c(\alpha )⩽c(\lambda^{⁎})$ and/or $d(\lambda )⩽d(\lambda^{⁎})$ is feasible for $\lambda^{⁎}$. This helps practical implementations since $c(\lambda^{⁎})$ are the maximum allowable costs for delivering a maximum amount of demands $d(\lambda^{⁎})$ so the analyst can know the optimal quantities to be sent to customers at a maximum allowable cost.

The optimal solution provides the amount of vehicles to be sent, the routes to cover all demands and crisp values for $c_{ij}(\alpha )\in {\overset{˜}{c}}_{ij}$ and $d_{i}(\lambda )\in {\overset{˜}{d}}_{i}$. The optimal $\lambda^{⁎}$ reaches equilibrium between delivery costs and satisfied demands which helps decision making since analysts are able to handle uncertain information and to provide routes to cover customers demands at a maximum allowable cost.

## Further topics

CVRPs with interval–valued capacities and fuzzy time windows are natural extensions to be solved with the proposed algorithm. Also Type-2 fuzzy numbers (see Figueroa-García [40]) can help to represent other uncertainties and fuzzy decision making techniques can help to improve its application in real world scenarios (see Rivera–Niquepa et al. [41] and Wu et al. [42]).

## Declarations

### Author contribution statement

Juan Carlos Figueroa Garcia: Conceived and designed the experiments; Wrote the paper. Carlos Franco: Analyzed and interpreted the data; Contributed reagents, materials, analysis tools or data. Johan Sebastian Tenjo Garcia: Performed the experiments; Contributed reagents, materials, analysis tools or data.

### Funding statement

This research did not receive any specific grant from funding agencies in the public, commercial, or not-for-profit sectors.

### Data availability statement

Data included in article/supp. material/referenced in article

### Declaration of interests statement

The authors declare no conflict of interest.

### Additional information

No additional information is available for this paper.

## Figures and Tables

**Table 3:** Fuzzy delivering costs and demands.

(*i*,*j*)	Set ${\overset{˜}{c}}_{ij}$	(*i*,*j*)	Set ${\overset{˜}{c}}_{ij}$	(*i*,*j*)	Set ${\overset{˜}{c}}_{ij}$	Set ${\overset{˜}{c}}_{ij}$	Demand	Value	
(1,2)	T(8,14,20)	(3,5)	T(18,22,26)	(5,12)	T(5,7,8)	(8,15)	T(9,15,19)	${\overset{ˇ}{d}}_{1}$	10
(1,3)	G(21,3,99)	(3,6)	T(12,16,20)	(5,13)	T(10,13,15)	(8,16)	G(32,6,24)	${\overset{ˇ}{d}}_{2}$	18
(1,4)	T(29,33,39)	(3,7)	G(11,2,70)	(5,14)	G(30,4,05)	(9,10)	T(15,17,23)	${\overset{ˇ}{d}}_{3}$	10
(1,5)	G(22,2,64)	(3,8)	T(7,9,13)	(5,15)	G(44,7,26)	(9,11)	T(14,15,19)	${\overset{ˇ}{d}}_{4}$	17
(1,6)	G(23,4,26)	(3,9)	T(10,12,15)	(5,16)	T(8,9,10)	(9,12)	G(32,4,48)	${\overset{ˇ}{d}}_{5}$	7
(1,7)	T(10,12,15)	(3,10)	G(15,2,70)	(6,7)	T(11,13,16)	(9,13)	G(17,2,64)	${\overset{ˇ}{d}}_{6}$	22
(1,8)	G(22,2,20)	(3,11)	T(8,11,12)	(6,8)	T(6,8,10)	(9,14)	T(8,10,15)	${\overset{ˇ}{d}}_{7}$	10
(1,9)	T(25,32,35)	(3,12)	G(29,7,11)	(6,9)	G(25,4,38)	(9,15)	G(31,6,20)	${\overset{ˇ}{d}}_{8}$	18
(1,10)	T(27,32,38)	(3,13)	G(19,2,38)	(6,10)	T(11,13,16)	(9,16)	T(18,23,27)	${\overset{ˇ}{d}}_{9}$	5
(1,11)	G(21,2,73)	(3,14)	T(7,9,13)	(6,11)	T(22,26,30)	(10,11)	T(22,25,26)	${\overset{ˇ}{d}}_{10}$	6
(1,12)	T(23,28,31)	(3,15)	G(24,5,64)	(6,12)	G(43,9,25)	(10,12)	G(44,7,92)	${\overset{ˇ}{d}}_{11}$	4
(1,13)	T(25,30,32)	(3,16)	T(20,23,26)	(6,13)	T(31,35,37)	(10,13)	T(26,31,34)	${\overset{ˇ}{d}}_{12}$	8
(1,14)	G(29,3,48)	(4,5)	T(17,21,23)	(6,14)	T(14,16,20)	(10,14)	T(5,7,10)	${\overset{ˇ}{d}}_{13}$	4
(1,15)	T(29,31,35)	(4,6)	G(31,4,34)	(6,15)	T(6,8,14)	(10,15)	G(16,1,76)	${\overset{ˇ}{d}}_{14}$	11
(1,16)	G(30,4,05)	(4,7)	G(25,6,00)	(6,16)	G(39,6,83)	(10,16)	G(37,9,25)	${\overset{ˇ}{d}}_{15}$	7
(2,3)	G(12,1,98)	(4,8)	T(15,23,28)	(7,8)	T(7,10,15)	(11,12)	T(14,19,22)	${\overset{ˆ}{d}}_{1}$	24
(2,4)	T(16,19,25)	(4,9)	T(6,8,11)	(7,9)	T(17,23,29)	(11,13)	T(8,10,12)	${\overset{ˆ}{d}}_{2}$	34
(2,5)	T(8,12,17)	(4,10)	T(18,24,26)	(7,10)	G(20,2,70)	(11,14)	G(18,4,23)	${\overset{ˆ}{d}}_{3}$	22
(2,6)	G(24,4,56)	(4,11)	T(8,12,14)	(7,11)	G(16,1,60)	(11,15)	G(34,5,78)	${\overset{ˆ}{d}}_{4}$	29
(2,7)	T(10,12,16)	(4,12)	G(25,3,13)	(7,12)	G(31,5,89)	(11,16)	T(9,13,20)	${\overset{ˆ}{d}}_{5}$	15
(2,8)	G(19,3,42)	(4,13)	T(7,9,10)	(7,13)	T(21,26,28)	(12,13)	T(14,16,35)	${\overset{ˆ}{d}}_{6}$	35
(2,9)	G(21,2,10)	(4,14)	G(17,2,55)	(7,14)	T(14,17,24)	(12,14)	G(37,4,81)	${\overset{ˆ}{d}}_{7}$	23
(2,10)	G(27,2,97)	(4,15)	G(37,7,77)	(7,15)	G(21,2,63)	(12,15)	T(45,51,68)	${\overset{ˆ}{d}}_{8}$	35
(2,11)	T(6,7,9)	(4,16)	G(16,2,24)	(7,16)	T(20,28,32)	(12,16)	T(8,10,12)	${\overset{ˆ}{d}}_{9}$	15
(2,12)	T(14,19,25)	(5,6)	G(36,6,12)	(8,9)	T(15,18,24)	(13,14)	G(24,4,44)	${\overset{ˆ}{d}}_{10}$	10
(2,13)	G(16,3,92)	(5,7)	G(24,3,36)	(8,10)	T(9,10,14)	(13,15)	G(43,6,88)	${\overset{ˆ}{d}}_{11}$	13
(2,14)	G(21,3,26)	(5,8)	T(27,30,36)	(8,11)	G(19,3,80)	(13,16)	T(5,6,8)	${\overset{ˆ}{d}}_{12}$	22
(2,15)	T(28,33,37)	(5,9)	T(24,26,28)	(8,12)	G(37,9,25)	(14,15)	T(14,21,23)	${\overset{ˆ}{d}}_{13}$	13
(2,16)	G(17,2,38)	(5,10)	G(37,5,18)	(8,13)	T(22,28,33)	(14,16)	G(30,6,75)	${\overset{ˆ}{d}}_{14}$	24
(3,4)	G(15,1,95)	(5,11)	G(12,2,82)	(8,14)	T(7,9,12)	(15,16)	G(47,8,23)	${\overset{ˆ}{d}}_{15}$	18

## Appendix A Fuzzy delivering costs and demands

Note that parameters *b* and *μ* corresponds to the deterministic costs of the original instance.
